# Revision Hip Arthroplasty Using a Modular Head–Neck Adapter System and a Ceramic Head: 5-Year Clinical and Radiographic Outcomes

**DOI:** 10.3390/jcm12144699

**Published:** 2023-07-15

**Authors:** Filippo Caternicchia, Valentina Fantoni, Andrea Poletto, Francesco Pardo, Francesco Castagnini, Francesco Traina

**Affiliations:** Orthopaedics-Traumatology and Prosthetic Surgery and Revision of Hip and Knee Implants, IRCCS Rizzoli Orthopaedic Institute, 40136 Bologna, Italy; filippo.caternicchia@ior.it (F.C.); valentina.fantoni@ior.it (V.F.); francesco.pardo@ior.it (F.P.); francesco.castagnini@ior.it (F.C.); francesco.traina@ior.it (F.T.)

**Keywords:** Bioball Merete, modular head–neck adapter, Delta ceramic head, revision, instability, offset

## Abstract

Introduction: A modular head–neck adapter system may help surgeons restore the proximal hip biomechanics in revision hip arthroplasty. However, the clinical and radiographic 5-year outcomes of the system are still scarcely reported. The aim of this study is the assessment of (1) complications and survival rates, (2) clinical and (3) radiological outcomes of the modular head–neck adapter system with a ceramic head in revision hip arthroplasty at 5 years. Methods: 32 revision hip arthroplasties using a modular head–neck adapter system and a ceramic head were retrospectively recorded. Preoperative demographic and implant features were collected. Clinical and radiographic outcomes, failures and reasons for re-revisions were recorded at the last follow-up. Results: A mean follow-up of 59.8 ± 26 months was achieved. The survival rate was 90.6% at 5 years. Post-operative complications occurred in nine cases (28.1%): two dislocations (6.2%), one aseptic cup loosening (3.1%) requiring re-revision, one (3.1%) persistent pelvic pseudotumor requiring embolization. No mechanical failures of the adapter or ceramic head occurred. The mean post-operative HHS score was 84.4 points. Thirteen cases (40.6%) showed optimal radiographic cup osseointegration, and 17 hips (53.1%) showed valid stem osseointegration. The mean post-operative femoral offset was 48.84 mm, larger than the contralateral side (*p* = 0.02). The post-operative lateralization of the hip joint was 38.09 mm, close to the contralateral side (*p* = 0.4). Conclusions: In revisions, the modular head–neck adapter system with ceramic head provided good clinical outcomes and acceptable radiographic reconstruction of hip biomechanics, with a survival rate of 87.9% at 5 years.

## 1. Introduction

Modular head–neck adapters have been introduced on the market as a modular hip joint solution for complex cases and unexpected situations in primary endoprosthesis and, mostly, revision hip arthroplasties. The system joins with the different Morse tapers of femoral stems, even retained ones, and allows the engagement of a new metal or ceramic head.

In revisions, the use of a modular head–neck adapter could restore the correct hip biomechanics through the intraoperative fine tuning of length, version or offset, aiming to avoid the risks of instability and unnecessary morbidity due to the removal of fully integrated components [1,2]. However, modularity carries adjunctive risks of failure, like breakage, dissociation, fretting, corrosion and consequent generation of adverse local tissue reactions. Similarly, the use of ceramic head in revisions has been demonstrated to reduce the rate of failures in comparison with metal balls [3]. However, ceramic heads, while providing minimal wear for the superior tribological properties, may incur specific fracture risks due to material brittleness [4].

**Figure 1:**
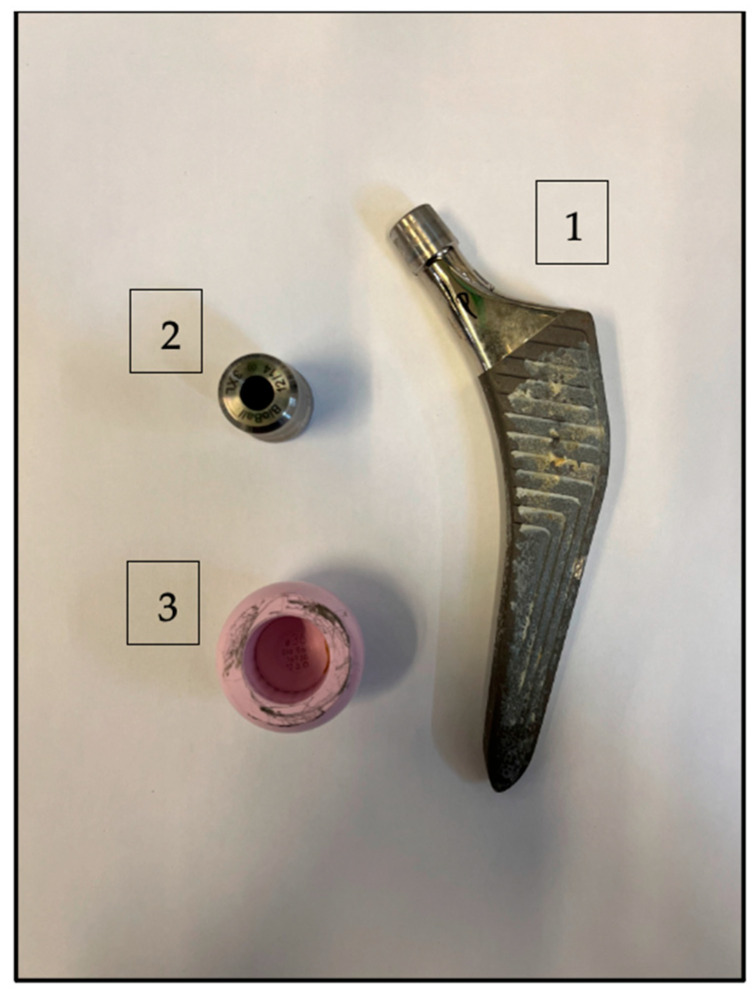
Implant overview: stem (1), modular head–neck adapter (2), ceramic head (3).

There is a paucity of literature reporting the 5-year outcomes of revisions with a modular head–neck adapter and ceramic head (Figure 1). In a registry report, the system with a ceramic head achieved a 5-year survival rate of 87.9%, with no mechanical failures: similar outcomes were achieved by titanium-sleeved ceramic heads in revisions, used as a control group. However, the clinical and radiographic outcomes of modular head–neck adapter and ceramic head in specific clinical case series were not described.

Thus, a consecutive case series of a modular head–neck adapter and a ceramic head was reviewed. The aims of this study were to assess the complications and the survival rates and the clinical and the radiographic outcomes (in particular, the reconstruction of proximal hip biomechanics) of modular head–neck adapter system with ceramic head in revision hip arthroplasty at 5 years. We hypothesized that a modular head–neck adapter system with a ceramic head in revisions could provide a 5-year survival rate of more than 90%, good clinical outcomes (Harris Hip score > 80 points), and effective offset and leg length restorations in most cases.

## 2. Materials and Methods

The institutional review board approved the study protocol 349/2021/Oss/IOR, 10 May 2021.

The hospital database was investigated about revision hips performed in our tertiary center from 2000 to 2017. Revision hips were prospectively evaluated using standard clinical and radiographic follow-ups as standard clinical practice. A consecutive series of these prospectively followed patients with revisions was retrospectively reviewed.

The inclusion criteria were: revision hip arthroplasty (rTHA) with the modular head–neck adapter system Bioball Merete (Medical GmbH, Berlin, Germany) and Delta ceramic head (Ceramtec, Plochingen, Germay), clinical chart availability and adhesion to post-operative clinical and radiographic follow-ups. Revision with implants other than the aforementioned ones, primary total hip arthroplasty (pTHA), lack of medical records and patients lost to follow-ups were excluded.

Out of 1712 revisions, 33 revisions were performed using a modular head–neck adapter system and a ceramic head. In one case, the medical chart was unavailable, and the patient was excluded. Thirty-two revisions in 32 patients matched the inclusion criteria and were included.

The population study included 22 females (68.8%), and 10 males (31.2%) with a mean age at revision surgery of 64.8 ± 8.9 years (range: 45–87 years). The ASA (American Society of Anesthesiology) score, assessing the fitness of candidates to surgery, and the Charlson comorbidity index, a comorbidity score predicting the general mortality rate, were reported [5,6]. The demographics, the ASA score and the Charlson comorbidity index score are detailed in Table 1.

All the revision surgeries were pre-operatively studied using standard pelvis radiographs and computer tomography (CT) scans extended from the fourth lumbar vertebra to the tibial plateaus. All the procedures were performed with the patient in supine decubitus, with a lateral approach. Primary implants were total hip arthroplasties, with a mean age at surgery of 64.8 ± 8.9 years (range: 45–87 years): 19 cases were first-time revisions (59.4%) and 13 (41.6%) were re-revisions. The bearing surfaces of the implants to be revised were: ceramic on polyethylene (CoPE) in 17 cases (53.1%), metal on polyethylene (MoP) in 12 cases (37.5%), ceramic on ceramic (CoC) in 2 cases (6.3%), metal on metal (MoM) in 1 case (3.1%).

The reasons for revision were: cup loosening in 16 cases (50%), polyethylene wear in 7 cases (21.9%), recurrent dislocations in 4 cases (12.5%), adverse reaction to metal debris in 2 cases (6.3%), Vancouver B2 periprosthetic fracture in 1 case (3.1%), and femoral neck stem fracture in 1 case (3.1%). In 26 cases (81.2%) major revisions were required: 23 cup revisions (71.9%), 2 stem revisions (6.3%) and 1 total hip revision (3.1%). Minor revisions with only modular part exchange were performed in six cases (18.8%): head exchange was performed only in two cases (6.25%).

Additional procedures, bone grafting and pseudotumor excision, were performed in three cases (9.4%).

The Merete^TM^ Bioball System is an implant available in different lengths (−3 mm; +21 mm), built in titanium alloy and compatible with different Morse tapers (11/12;12/14;14/16). It is available in neutral and 7.5° offset. Modular heads are available in metal or ceramic material from 28 to 36 mm in diameter. Only Delta (CeramTec, Plochingen, Germany) heads were adopted.

This modular head adapter system was adopted when additional length, offset or version correction was needed, no titanium sleeved heads could be implanted, and well-fixed component revision was undesirable. The final modular component choice was made after multiple intra-operative trails, assessing implant stability and soft tissue tensioning. The most frequently used adapter in this study was 2XL (+10.5 mm) accounting for 28.1%, followed by XL (+7 mm) and L size (+10.5 mm), both accounting for 18.8%. Moreover, both M (0 mm) and 3XL (14 mm) sizes were adopted in 15.6% of cases, while one S size adapter (−3 mm) only was used (3.1%). Offset adapter was implanted in two patients (6.25%). The 12/14 taper adapters were used in 24 cases (75%), and 14/16 tapers in 6 cases (18.8%). Among the 12/14 tapers, two (6.25%) were with offset correction. A 32 mm femoral head was implanted in 12 patients (52%), in eight patients (35%) a 36 mm head was adopted, and 28 mm balls were used in three patients (13%). The bearing surfaces were CoPE in 21 cases (65.6%) and CoC in 10 cases (31.3%).

Perioperative complications were recorded and classified according to Clavien–Dindo classification [7]. Implant survival was determined and reasons for re-revision were listed. Patients were clinically and radiographically evaluated at one month, three months and then annually. Clinical assessment was conducted using Harris Hip Score (HHS) [1]. The radiographic evaluation was conducted by the first author: osteointegration, radiolucency, osteolysis or migration were assessed. Cup osseointegration according to Moore Criteria and stem bony ingrowth according to Engh criteria were evaluated in the last available X-rays [8,9]. Limb-length discrepancy (LLD), acetabular offset, femoral offset, acetabular cup inclination and vertical offset were assessed in the last available pelvis X-rays after appropriate magnification. A comparison between femoral offset and acetabular offset of the operated side with contralateral side was performed.

### Statistical Analysis

The quantitative data were expressed as average values, standard deviations, and ranges of minimum and maximum. The qualitative data were expressed as frequencies and percentages. The survival of the implants was calculated and plotted according to the Kaplan–Meier method, with removal or change of any component as the endpoint.

The implants were followed until the last date of observation (date of death or 31 January 2023). Data analysis was undertaken using the chi squared test. The statistical analysis was performed using SPSS 14.0 for Windows, version 14.0.1 (SPSS, Chicago, IL, USA). A *p*-value = 0.05 was considered statistically significant.

## 3. Results

A mean follow-up of 59.8 ± 26 months was achieved. No cases were lost to follow-up.

Post-operative complications were registered in nine cases (28.1%). Nineteen patients (59.3%) reached a peri operative Clavien–Dindo grade of 2, ten patients (31.2%) reached a Clavien–Dindo grade of 1 while three patients (9.3%) were classified with a Clavien–Dindo grade of 3. We recorded recurrent dislocation in two cases (6.2%), fair Trendelenburg gait in one case (3.1%), aseptic cup loosening and proximal femoral osteolysis with pseudotumor (without stem loosening) in one case (3.1%), one case (3.1%) of persistent pelvic pseudotumor, three cases (9.4%) of post-operative anemia, one case (3.1%) of lower limb phlebitis.

The aseptic loosening of the cup underwent a cup re-revision and a new excision of the pseudotumor. One case of dislocation was conservatively managed. The second case of dislocation was no longer eligible for a new surgery (cup revision for excessive combined anteversion) due to severe comorbidities and was treated conservatively. No more dislocations occurred in both the cases.

The persistent post-operative pseudotumor was managed with embolization. Post-operative anemia was managed with blood transfusion in two cases while embolization was required in one case.

**Figure 2:**
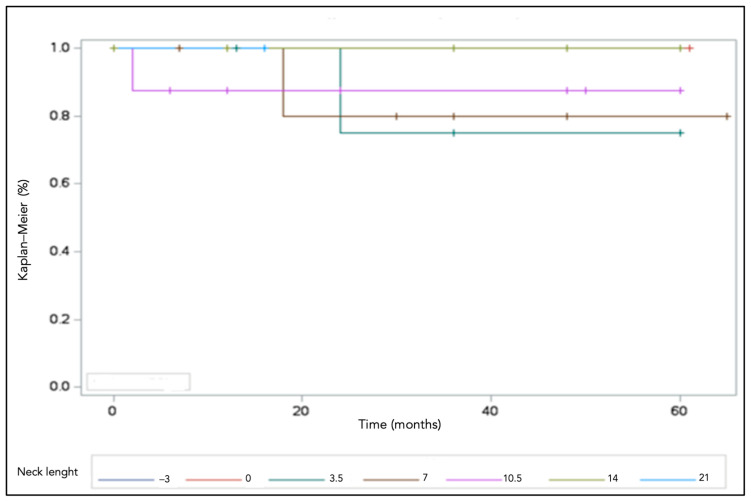
The survival of the implants was 87.9% at 5 years.

No mechanical failures of the modular adapter and head occurred. The global implant survival rate was 87.9% (95% CI 81.3–94.5) at a 5-year follow-up (Figure 2).

**Figure 3:**
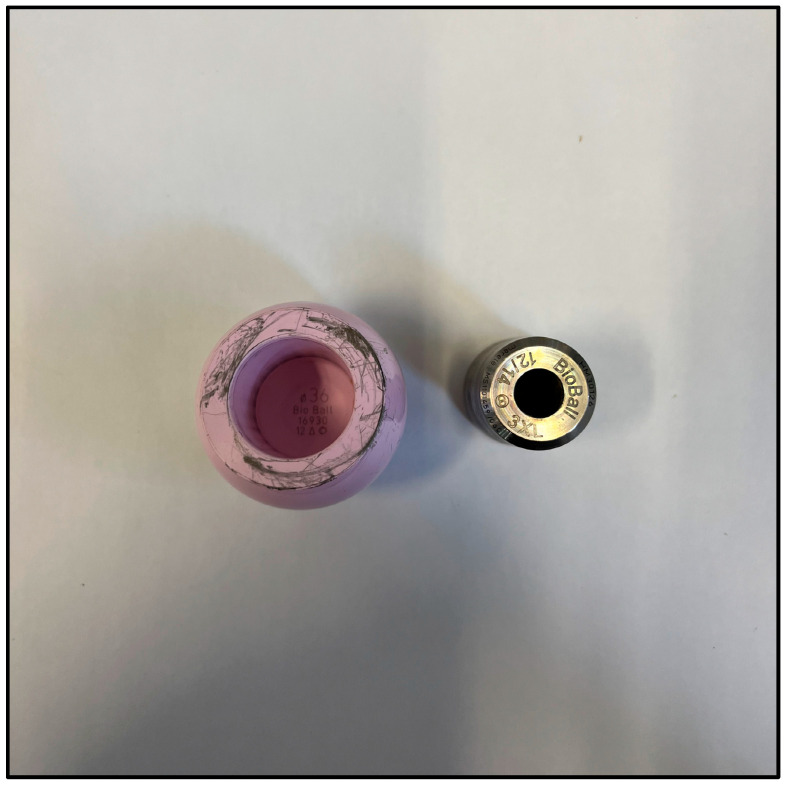
No signs of corrosion of implants breakage were recorded.

Explanted components did not show any macroscopic signs of mechanical failures, (Figure 3), including fretting corrosion or implant breakage.

The mean HHS score increased form a pre-operative value of 46.9 ± 11.1 to the post-operative value of 84.4 ± 9.3 points (*p* = 0.01).

Ten cases (31.2%) presented with excellent results (HHS > 90 points), twelve cases (37.5%) with good results (HHS 80–89 points), seven cases (21.9%) with fair results (HHS 70–79 points), three patients (9.4%) with poor results (HHS < 70 points).

**Figure 4:**
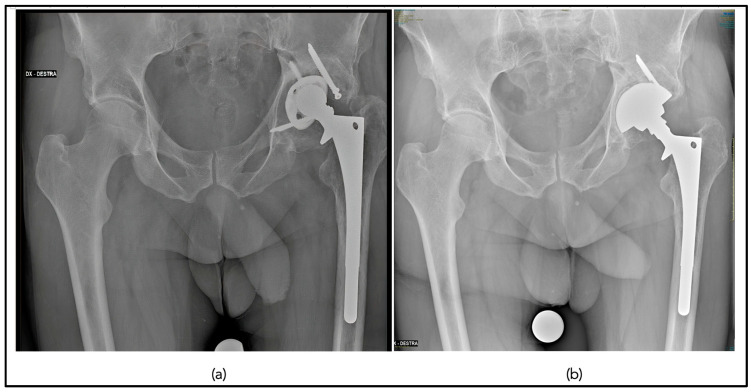
Pre-operative anteroposterior pelvic radiograph demonstrating cup mobilization and polyethylene liner wear (**a**); post-operative anteroposterior pelvic radiograph at 5 years following stem retention use of the Bioball head–neck adaptor and revision of the acetabular component (**b**).

No cup or stem components showed progressive radiolucent lines or osteolysis at the last follow-up (Figure 4).

No cup or stem components showed progressive radiolucent lines or osteolysis at the last follow-up, Image 3. No component migration occurred. Cups showed good signs of radiographic osseointegration (>3 Moore criteria for bony ingrowth) in 13 cases (40.6%). Stems showed good signs of bony ingrowth in 17 hips (53.1 %) with a mean Engh criteria of 11.6 ± 7.5. The mean post-operative femoral offset (48.8 mm ± 10.33) was larger than the contralateral side (45 mm ± 7) (*p* = 0.02). The post-operative acetabular offset was 38 mm ±6.4 compared to 39.3 mm ± 7.2 of the contralateral side (*p* = 0.08). The mean LLD was 8.7 mm ± 8.1. The mean post-operative vertical offset was 23 mm ± 4.6 compared to 22.79 ±4.7 of the contralateral side (*p* = 0.06). The post-operative acetabular cup inclination was 42.7° ± 8.2 compared to 42.4° ± 7.1 (*p* = 0.07).

## 4. Discussion

This modular system achieved a satisfying survival rate (87.9% at 5 years) with good clinical outcomes (two thirds with good-to-excellent HHS score) and acceptable restoration of post-operative radiographic femoral offset compared to the contralateral side.

We reported a survival rate of 87.9%. This outcome is similar to and supported by previous studies in the literature. Hoberg et al. [10] showed promising results using the same system with a survival rate of 92.8% at 8.17 years (mixed head materials, ceramic and metal heads). Pardo et al. [11], in a registry study analyzing 354 modular head adapters coupled with ceramic heads, showed a survival rate of 87.8% at 5 years and 85.7% at 10 years, close to our case series. Offset and skirted devices did not impact the survival rates. The modular adapter cohort showed a similar survival rate when compared to ceramic heads with factory assembled titanium sleeve.

In the present case series, a global rate of complications of 28.1% was reported: the dislocation rate was 6.2%. Recurrent dislocations after revisions are well-known complications, occurring in up to 30% of the cases and depending on multiple risk factors (like surgical approach, soft tissue management, implant features, incomplete proximal biomechanics restoration) [12,13]. The present case series achieved a dislocation rate of 6.2%, similar to the 6.3% reported by Dabis et al. [14] in 32 revisions with the modular head–neck adapter. Nonetheless, this dislocation rate can be considered favorable when compared to the current literature about revision hips (around 9.8%) [12,13].

The failure of the neck adapter could be a potential reason for concern in revisions. In our series, no mechanical failure of the devices occurred: no dissociation between the sleeve and the stem and no breakage of the components (adapter and ceramic head). This result confirmed that mechanical failures of the modular head adapter system are anecdotical. Pardo et al. did not report any mechanical failure in a cohort of 354 modular adapters with ceramic head [11].

Supported by Hoberg at all [10], no macroscopic fretting corrosion or osteolysis of femoral implant was observed in our series after a follow up of 5 years. These results are probably due to the benefit of the titanium adapter alloy and ceramic head [14]. The solid outcomes of ceramic bearings in terms of minimal wear and reduction of fretting corrosion were reported in laboratory studies [15,16]. However, this case series cannot exclude excessive titanium ion release or subclinical fretting corrosion.

The modular head adapter generally achieved satisfying results at 5 years with a mean HHS score of 84 ± 9.3 points. Clinical outcomes were excellent in 31.2% of cases. These results are similar to the results supported by Jack et al. [4] that showed a mean HHS of 91 in 165 partial acetabular revisions with retained stems. Clinical comparisons to other case series with modular adapter are challenging, as a wide range of post-operative HHS scores, ranging from 50 to 90 and more, was reported [10].

We achieved an improvement in post-operative radiographic femoral offset compared to the contralateral side (48.8 ± 10.3 mm, *p* value = 0.02). Theoretically, using the longest adapters with offset configuration increases the possible mechanical failure of the system and may influence implant stability due to the skirted configuration. We did not observe a negative effect with longer necks and skirted configurations, similarly to Woelfle et al. and Pardo et al. [11,17].

In this study, we achieved a good restoration of hip biomechanics compared to the contralateral side. The valid restoration of proximal hip biomechanics is a common finding in the literature describing the modular head–neck adapter [18]. The post-operative femoral offset was increased in the present case series, with the aim to improve abductor lever arm and implant stability. In musculoskeletal models of THA patients performing different activities at risk of dislocations, +5 and +9 mm offset implants, with an associated increase in capsular tension, increased the resistive moment during dislocation of 78.1% and 121% with respect to neutral offset implants [19].

This article has many limitations. First, the small case series, the retrospective design and the lack of control group are the main drawbacks. Only a 5-year follow-up was achieved. Metal ion levels were not assessed, even if no relevant signs or symptoms or adverse local tissue reactions could be identified at the final follow-up. Only a macroscopic analysis of the explanted components was conducted, with no evidence of mechanical failure: a proper retrieval analysis was not conducted, also because of the limited post-operative follow-up of the re-revised cases. On the other hand, this is one of the largest clinical studies with a homogeneous case selection providing mid-term clinical outcomes of modular head–neck adapter system with a ceramic head in rTHA.

In conclusion, this versatile system achieved a global survival rate of 87.9% at 5 years with reasonable rates of complications and dislocations rates. No mechanical failures occurred. One third of the patients achieved excellent clinical outcomes. Many cases achieved a very good reconstruction of the proximal hip biomechanics, with increased offset. Further large long term multi-center studies are necessary for evaluating important data about this modular adapter system concerning mechanical failure, wear and corrosion.

## Figures and Tables

**Table 1:** The demographics, the ASA score and Charlson comorbidity index of the selected cohort. The female population was the most represented; overweight patients represented almost half of the population. The most common score on the Charlson Comorbidity index was 3–4 with an estimated 10-year survival of 53 to 77%.

Demographics	Data
Mean age at pTHA, standard deviation in years (range)	51.5 ± 12.3 (29–79)
Mean age at rTHA, standard deviation in years (range)	64.8 ± 8.9 (45–87)
Mean weight (kg) ± standard deviation	76.4 ± 22.7
Female sex, *n* (%)	2 (68.8)
Male sex, *n* (%)	10 (31.2)
Mean BMI, kg/m^2^ (range)	27.60 ± 4.4 (19.6–37)
Overweight patients	14 (43.75)
Obese patients	7 (21.87)
ASA score, *n* (%)	
1 and 2	26 (81.2)
3 and 4	6 (18.7)
Missing data	0 (0)
Charlson Comorbidity index, *n* (%)	
0–2	12 (37.5)
3–4	14 (43.7)
5 or more	6 (18.75)
Smoking patients, *n* (%)	6 (18.8)

## Data Availability

The data presented in this study are available on request from the corresponding author. The data are not publicly available due to privacy reasons.
